# A Wearable Multi-Modal Digital Upper Limb Assessment System for Automatic Musculoskeletal Risk Evaluation

**DOI:** 10.3390/s23104863

**Published:** 2023-05-18

**Authors:** Abdullah Tahir, Shaoping Bai, Ming Shen

**Affiliations:** 1 Department of Electronic Systems, Aalborg University, 9220 Aalborg, Denmark; atah@es.aau.dk (A.T.); mish@es.aau.dk (M.S.); 2 Department of Mechanical, Mechatronics, and Manufacturing Engineering, University of Engineering & Technology Lahore, Faisalabad Campus, Faisalabad 38000, Pakistan; 3 Department of Materials and Production, Aalborg University, 9220 Aalborg, Denmark

**Keywords:** digital upper limb assessment (DULA), rapid upper limb assessment (RULA), musculoskeletal disorder (MSD), MSD prevention and intervention, multi-modality wearable sensors

## Abstract

Continuous ergonomic risk assessment of the human body is critical to avoid various musculoskeletal disorders (MSDs) for people involved in physical jobs. This paper presents a digital upper limb assessment (DULA) system that automatically performs rapid upper limb assessment (RULA) in real-time for the timely intervention and prevention of MSDs. While existing approaches require human resources for computing the RULA score, which is highly subjective and untimely, the proposed DULA achieves automatic and objective assessment of musculoskeletal risks using a wireless sensor band embedded with multi-modal sensors. The system continuously tracks and records upper limb movements and muscle activation levels and automatically generates musculoskeletal risk levels. Moreover, it stores the data in a cloud database for in-depth analysis by a healthcare expert. Limb movements and muscle fatigue levels can also be visually seen using any tablet/computer in real-time. In the paper, algorithms of robust limb motion detection are developed, and an explanation of the system is provided along with the presentation of preliminary results, which validate the effectiveness of the new technology.

## 1. Introduction

Physical workload assessment is critical but complex and multi-dimensional, which requires careful consideration of various physical workload modalities, e.g., intensity, duration, frequency, and variability, together with environmental and individual factors, e.g., temperature, lighting conditions, age, sex, and physical fitness [1]. Workers in factories, individuals performing activities of daily life (ADL) with poor posture, and even computer and mobile phone users are prone to develop MSDs [2]. MSDs include temporary or permanent injuries in the inner body parts, e.g., muscles, tendons, joints, and spinal disks. Industrial workers often have to perform repetitive movements, heavy lifting, and body twisting, which can lead to a variety of problems, e.g., chronic pain, decreased mobility, and MSDs. Such jobs require workers to work in conditions that are not conducive to good posture and body alignment [3], hence carrying high risks of developing MSDs. A recent survey for working conditions in Europe indicates that 42% of European workers complain of pain around the neck and upper limbs [4].

To ensure the safety of workers, it is important to assess the ergonomic risks associated with these tasks and implement controls and interventions to improve working conditions. Several risk assessment methods have been developed, such as rapid entire body assessment (REBA) [5], novel ergonomic postural assessment (NERPA) [6], strain index (SI) [7], Ovako working posture analyzing system (OWAS) [8], and RULA [9]. Of them, RULA is the most widely used subjective tool to quickly assess the ergonomic risk factors associated with upper limbs, incorporating both muscle utilization and limb postures. Basahel et al. used RULA to assess ergonomic risk factors associated with lifting and pulling tasks in an industrial environment [10]. In another study, a modified RULA method was proposed for two posture risk quantification methods, namely event-based and time-based, for upper-extremity MSDs [11]. Additional similar works using RULA and its modified forms to assess ergonomic risk factors associated with industrial workers can be found in references [12,13]. Since RULA requires deployment in the field, therefore these non-automatic methods require tedious setup and a medical expert to continuously observe workers’ body movements. To empower devices to take on the burden from humans, body-mounted sensors can be used for computing RULA scores automatically, e.g., electromyograph (EMG) based methods [14,15,16] and inertial-sensor-based methods [17,18,19]. However, it is essential to note that EMG-based methods have higher costs and longer deployment times. The past works utilizing inertial-based methods do not explicitly provide kinematic details and use graphic software, e.g., CATIA or AnyBody, to show limb movements, which put a limitation on the design and deployment of an automatic RULA assessment system in the form of an embedded wearable device.

As a graphical assessment technique, a digital human model (DHM) can be used to visualize and analyze the movements of limbs and human posture in computer software [20,21]. Such techniques are particularly useful to identify and mitigate potential risks before designing a product or workstation. This technology is suitable for early ergonomic analysis but cannot be utilized in real-time, as performing a certain task is majorly dependent upon an individual’s preference. Therefore, the utilization of muscles or body posture would be different for the same task from time to time and person to person. Hu et al. worked on predicting the real-world ergonomic measurements by simulating the scenario in a virtual environment (VE) while a subject did the movements in a real environment [22]. Nevertheless, VE-based methods can only be deployed and tested in a laboratory setup. Another mode of risk assessment is based on video recording [23,24,25]. Optical assessment methods are tedious to set up with the constraint of having direct sight of the worker. Other assessment methods, e.g., questionnaires and interviews [26,27], limit their practicality due to the substantial need for human resources, and such methods can be biased.

For the people working in environments where the setup is already installed, non-obstructive and intrinsic or body-mounted sensors, e.g., EMG, inertial sensors, gyroscope, and gravimeters, can be used for recording and analyzing motion data, and further for ergonomic risk assessments [17,18,19,28]. Padilla et al. designed a web-based risk-factor assessment system using inertial sensors and displayed limb movements [29], but they did not provide any details for limb kinematics. Employing graphic software to display limb movements is common but not very helpful in assessing working conditions due to the lack of kinematic information or evaluating exoskeleton design, particularly passive exoskeletons [16,30] in industrial usages. A comparison of past works is listed in Table 1.

In this study, a multi-modal digital upper limb assessment system—DULA—is presented along with robust kinematics, which continuously tracks and records muscle activities and limb motions to digitally fill the RULA chart and automatically generate risk levels. DULA consists of multi-modal sensor bands having wireless sensors interfaced with a mobile or tablet application. Two modalities, (i) limb posture in space using IMUs, and (ii) muscle activity through a force myograph (FMG), are devised for the proposed system. A robust kinematic model is developed to accurately determine upper limb motion. Furthermore, the data from these wearable sensors is continuously recorded and stored in Google Firebase, which can be retrieved for visualization, examination, and further analysis by a medical expert. The contributions of this work are:

1. Multi-modal wireless wearable sensors are used for digitally computing RULA scores.
2. A robust kinematic model has been developed for upper limbs motion tracking.
3. Digital implementation of the new method has been validated by comparison with an online RULA calculator.

The balance of the paper is organized as follows: Section 2 describes RULA, kinematics to detect arm posture, frame transformations, and load identification. Hardware and software implementation is presented in Section 3. Results are presented in Section 4 and discussed in Section 5. Finally, conclusions are drawn in Section 6.

## 2. DULA Method

The DULA system utilizes battery-powered wireless multi-modal sensor bands, which are wirelessly connected to a mobile application through Bluetooth. As will be presented in Section 3, each sensor band is equipped with an IMU and force-sensitive resistors (FSRs) for FMG, which are primarily used for ergonomic risks analysis, whereas gyroscopes and gravimeters are used to set up coordinate frames with the upper limbs for kinematic analysis. The system automatically generates the RULA score, which will be henceforth referred to as the DULA score.

### 2.1. RULA

**Figure 1:**
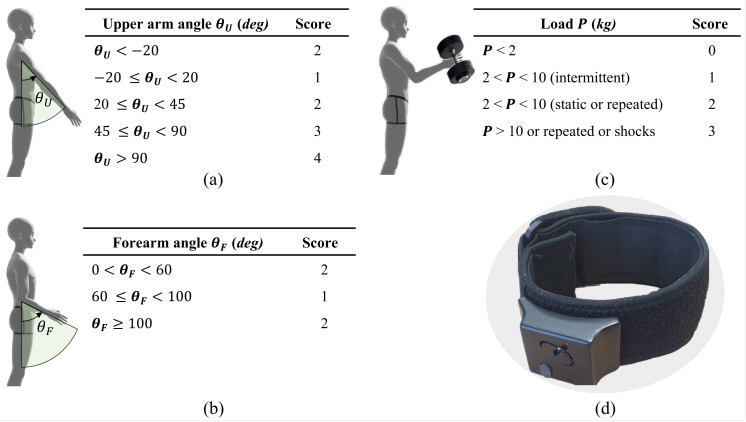
DULA diagram for ergonomic assessment. (**a**) Upper arm postures and corresponding scores, (**b**) forearm postures and corresponding scores, (**c**) load being lifted and corresponding scores, (**d**) wearable sensor band.

DULA is a digital implementation of RULA. We will, therefore, briefly describe the RULA method for completeness’ sake. RULA is a subjective observation method of posture analysis to assess the risks of developing MSDs for individuals who perform physical tasks using their upper extremities. Illustrations for different upper limb postures, the load being lifted, and associated numerical scores are shown in Figure 1 [9]. The DULA system finds the orientation of the upper arm and forearm and the load being lifted, assigns the corresponding scores, and yields the aggregated score. The aggregated score has the range of 1–9, which determines the overall risk of MSDs for upper limbs [9] and the corresponding action level as shown in Table 2. A lower RULA score corresponds to a lower risk of developing MSDs indicating that work posture is good enough. Hence, no change in the working scheme is required. In contrast, a higher RULA score corresponds to a severe risk of developing MSDs, and therefore, the working scheme should be immediately changed.

### 2.2. Kinematics for DULA

DULA, i.e., automatic computation of RULA score, requires first determining the accurate forearm and upper arm orientation. IMUs in the sensor band are primarily used for this purpose. However, raw data from IMUs do not provide any useful kinematic information without the attachment of reference coordinate frames on the upper arm and forearm; doing so will provide a coordinate reference to measure the limb’s orientation. Setting up the coordinate frame is explained in Section 2.2.1 while frame transformations of the upper arm and forearm movements with respect to the attached coordinate frames are explained in Section 2.2.2 and Section 2.2.4.

#### 2.2.1. Reference Coordinate Setup

**Figure 2:**
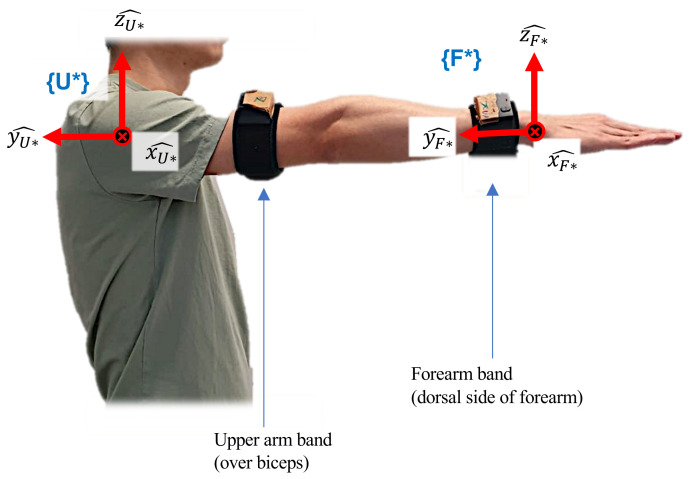
Reference coordinate frames setup for the forearm $\left\{F^{*}\right\}$ and the upper arm $\left\{U^{*}\right\}$.

The reference coordinate frames for the forearm and upper arm are shown in Figure 2. To attach a frame to the forearm, a sensor band is worn on the dorsal side of the arm, i.e., near the wrist. A certain posture and a certain movement are performed so that a coordinated setup can be established. Each course of action identifies one unit vector of the forearm as each movement projects one unit vector of the forearm reference frame in the coordinate frame identified by the current orientation of IMU referred to as {I}. The cross-multiplication of two orthogonal unit vectors from the two actions yields a third unit vector or axis and completes the procedure. The right forearm frame is settled with the following steps:

1. Stretch the arm forward while the palm is facing downwards. This is considered the right arm base posture.
2. Record the gravity vector *g* at base posture, upon which the $\hat{z_{F^{*}}}$ for the forearm frame is determined.
  (1) $$\hat{z_{F^{*}}}=-\frac{g}{∥g∥}.$$
3. Perform supination motion and record angular velocity ω during the motion. The $\hat{y_{F^{*}}}$ for the forearm frame is hence obtained as:
  (2) $$\hat{y_{F^{*}}}=-\frac{\omega }{∥\omega ∥}.$$
4. The cross product of $\hat{y_{F^{*}}}$ and $\hat{z_{F^{*}}}$ results in $x_{F^{*}}$ for the forearm frame. However, it is not humanly possible to have $\hat{y_{F^{*}}}$ and $\hat{z_{F^{*}}}$ exactly perpendicular to each other, hence, $x_{F^{*}}$ is normalized.
  (3) $$x_{F^{*}}=\hat{y_{F^{*}}}\times \hat{z_{F^{*}}},$$
  (4) $$\hat{x_{F^{*}}}=\frac{x_{F^{*}}}{∥x_{F^{*}}∥}.$$
5. Finally, $\hat{z_{F^{*}}}$ is obtained to complete right hand coordinate frame convention.
  (5) $$\hat{z_{F^{*}}}=\hat{x_{F^{*}}}\times \hat{y_{F^{*}}}.$$
6. Thus, the computed rotation matrix belongs to a special orthogonal group $SO\left(3\right)$. The forearm reference frame in the IMU frame has the following form:
  (6) $${}_{I}^{F^{*}}R=\left[\begin{array}{ccc} \hat{x_{F^{*}}} & \hat{y_{F^{*}}} & \hat{z_{F^{*}}} \end{array}\right].$$

The method to attach a reference coordinate frame on the right upper arm follows the same steps as the forearm, except that the sensor band is worn on the biceps muscles. The same steps are repeated for the left forearm and left upper arm; however, for the left limbs, pronation motion will be performed as in Step 3, and the angular velocity direction is taken positively.

#### 2.2.2. Frame Transformations

**Figure 3:**
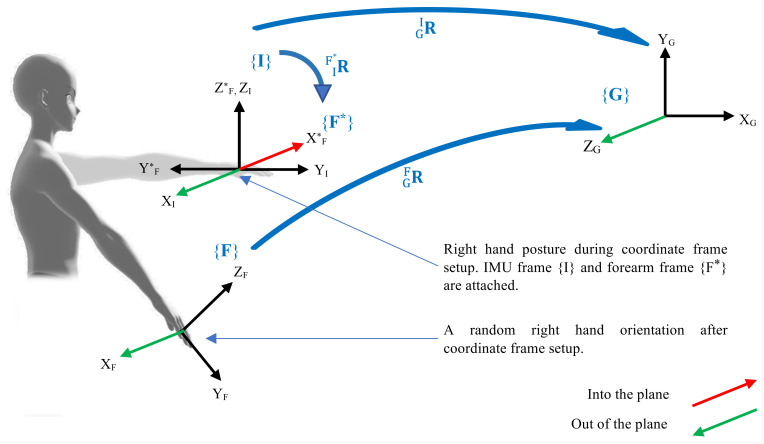
Frame setup for the kinematics of arm motion. The coordinate frames are shown in curly braces, whereas rotation matrices adjacent to arrows connecting two frames represent their transformation. {G} is the global reference frame. $\left\{F^{*}\right\}$ and {I} are the forearm reference frame and IMU frame, respectively, and they are attached to the initial configuration of the forearm. Frame {F} moves with the forearm.

A robust frame transformation methodology is developed, which provides the limbs orientations with respect to corresponding reference coordinate frames. A scenario, as shown in Figure 3, is presented for the right forearm, where the frames, shown in curly braces, are randomly oriented to formulate the mathematical expression of the rotation matrix to represent the orientation of the forearm {F} with respect to the forearm reference frame $\left\{F^{*}\right\}$. The matrix R represents a rotational transformation from the frame in the subscript to the frame in superscript, whereas the subscripts under the axes identify the frame they belong to.

Consider the posture when the sensor band is worn on the forearm. Raw data from the IMU represents the forearm orientation in global reference frame ${}_{G}^{F}R$. A reference frame $\left\{F^{*}\right\}$ is attached to the forearm according to the method described in Section 2.2.1. The objective is to find ${}_{F^{*}}^{F}R$, however, it should be noted that $\left\{F^{*}\right\}$ does not necessarily exhibit the same orientation as that of {G}. Furthermore, the reference frame $\left\{F^{*}\right\}$ constitutes a new coordinate system. Hence, the objective is to find the forearm frame {F} orientation which is defined with respect to {G}, in a new coordinate system established by $\left\{F^{*}\right\}$. The method is described as follows:

1. Record IMU orientation ${}_{G}^{I}R$ when the arm is in the base posture.
2. ${}_{I}^{F^{*}}R$ is computed according the method in Section 2.2.1.
3. ${}_{G}^{F}R$ is continuously acquired from the IMU sensor in real-time.
4. To find the forearm orientation with respect to IMU frame, i.e, ${}_{I}^{F}R$, the following equations are presented:
  (7) $${}_{G}^{F}R={\;}_{G}^{I}R{\;}_{I}^{F}R,$$
  (8) $${}_{I}^{F}R=\;{\left({}_{G}^{I}R\right)}^{T}{\;}_{G}^{F}R.$$
5. Finally, we find the orientation of the forearm with respect to the forearm reference frame $\left\{F^{*}\right\}$,
  (9) $${}_{F^{*}}^{F}R=\;{\left({}_{I}^{F^{*}}R\right)}^{T}{\;}_{I}^{F}R{\;}_{I}^{F^{*}}R.$$

The same methodology is applied to find upper arm transformation to have a rotation matrix describing upper arm {U} orientation with respect to upper arm reference frame $\left\{U^{*}\right\}$.

#### 2.2.3. Upper Arm Orientation

The rotation matrix defining upper arm orientation with respect to the upper arm reference frame is considered to be formed by the upper arm extension/flexion $\psi_{u}$, abduction/adduction $\theta_{u}$, and internal/external rotation $\psi_{u}$. Mathematically:

(10) $${}_{U^{*}}^{U}R=\;R_{XZ^{\prime }Y^{\prime \prime }}=\;R_{X}\left(\phi_{u}\right)\;R_{Z^{\prime }}\left(\theta_{u}\right)\;R_{Y^{\prime \prime }}\left(\psi_{u}\right)\;=\left[\begin{array}{ccc} r_{11} & r_{12} & r_{13}\\ r_{21} & r_{22} & r_{23}\\ r_{31} & r_{32} & r_{33} \end{array}\right],$$

where, starting with a frame coincident with upper arm reference frame $\left\{U^{*}\right\}$, $R_{X}$, $R_{Z^{\prime }}\left(\theta_{u}\right)$, and $R_{Y^{\prime \prime }}\left(\psi_{u}\right)$ represent three consecutive rotations following XZY convention. The angles $\phi_{u}$, $\theta_{u}$, and $\psi_{u}$ are obtained as:

(11) $$\phi_{u}=\arctan\left(r_{32},r_{22}\right),$$

(12) $$\theta_{u}=\arcsin\left(-r_{12}\right),$$

(13) $$\psi_{u}=\arctan\left(r_{13},r_{11}\right).$$

#### 2.2.4. Forearm Orientation

To compute the RULA score for a forearm, the relative rotation of the forearm with respect to upper arm is required. However, the orientations of the forearm and upper arm are defined in $\left\{F^{*}\right\}$ and $\left\{U^{*}\right\}$, respectively, while both constitute different coordinate systems. Therefore, we map the forearm orientation in the coordinate frame $\left\{U^{*}\right\}$ and then find the relative rotation.

**Figure 4:**
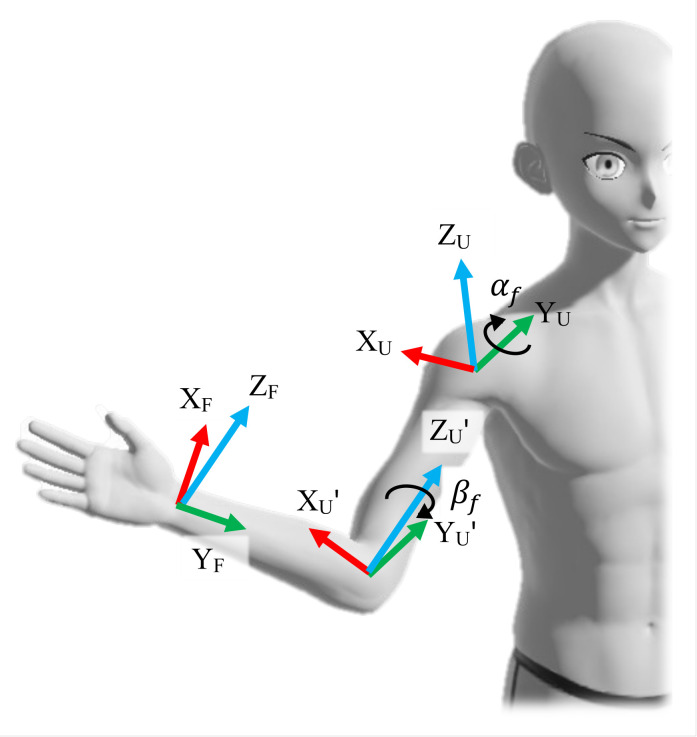
Moving from the upper arm frame to the forearm frame, when forearm orientation is already defined with respect to frame $\left\{U^{*}\right\}$.

1. Find the orientations of upper arm base frame $\left\{U^{*}\right\}$ and forearm base frame $\left\{F^{*}\right\}$ with respect to global reference frame {G} as:
  (14) $${}_{G}^{F^{*}}R={\;}_{G}^{I_{f}}R{\;}_{I_{f}}^{F^{*}}R,$$
  (15) $${}_{G}^{U^{*}}R={\;}_{G}^{I_{u}}R{\;}_{I_{u}}^{U^{*}}R,$$
  where $I_{u}$ and $I_{f}$ are upper arm IMU and forearm IMU frames, respectively.
2. Compute the relative rotation $R_{1}$ between both frames $\left\{F^{*}\right\}$ and $\left\{G^{*}\right\}$:
  (16) $${}_{G}^{F^{*}}R\;R_{1}={\;}_{G}^{U^{*}}R,$$
  (17) $$R_{1}={\left({}_{G}^{F^{*}}R\right)}^{T}{\;}_{G}^{U^{*}}R.$$
3. The forearm orientation {F} defined in $\left\{F^{*}\right\}$ is mapped in the upper arm reference frame $\left\{U^{*}\right\}$ using similarity transformation technique:
  (18) $${}_{U^{*}}^{F}R=R_{1}^{T}{\;}_{F^{*}}^{F}R\;R_{1}.$$
4. Since both forearm and upper arm orientations are now established in the same coordinate system defined by $\left\{U^{*}\right\}$, the relative rotation matrix $R_{2}$ is found as:
  (19) $${}_{U^{*}}^{U}R\;R_{2}{=}_{U^{*}}^{F}R,$$
  (20) $$R_{2}={\left({}_{U^{*}}^{U}R\right)}^{T}{\;}_{U^{*}}^{F}R.$$
5. The forearm orientation with respect to the upper arm is considered to be formed by the combination of two rotations, the forearm extension/flexion $\alpha_{f}$ about the current Y, and the forearm pronation/supination $\beta_{f}$ about the current Z axis.
  (21) $$R_{2}=R_{{YZ}^{\prime }}=\;R_{Y}\left(\alpha_{f}\right)\;R_{Z^{\prime }}\left(\beta_{f}\right)=\left[\begin{array}{ccc} s_{11} & s_{12} & s_{13}\\ s_{21} & s_{22} & s_{23}\\ s_{31} & s_{32} & s_{33} \end{array}\right].$$
  The composition of orientation is illustrated in Figure 4. Inverse kinematics is used to find the forearm angles:
  (22) $$\alpha_{f}=\arctan\left(s_{21},s_{22}\right),$$
  (23) $$\beta_{f}=\arctan\left(s_{13},s_{33}\right).$$

### 2.3. Load Identification

FMG is a technology that can measure changes in muscle volume during limb motion, which arise from muscle contractions and expansions. An FSR is a special resistor that exhibits a change in resistance when a force is applied to it. Therefore, FMG is primarily done using FSRs, and each sensor band is equipped with eight FSRs. Upper limb muscles hold the weight carried by an individual since the sensor band is worn over bicep muscles; hence the change in measured resistance corresponds to the muscle activity, which is interpreted as force being exerted or load being lifted by an individual. Various research works are available to identify payload using FMG [31,32]. When the sensor band is worn over the upper arm, the maximum value for each FSR channel is recorded by intense elbow flexion, later used to normalize FSR data. Normalized FSR values for 2 kg and 10 kg loads, as two thresholds for RULA, are important according to Figure 1c and are marked experimentally to identify the load category. Finally, the identified load is scored according to Figure 1c.

## 3. DULA System Development

**Figure 5:**
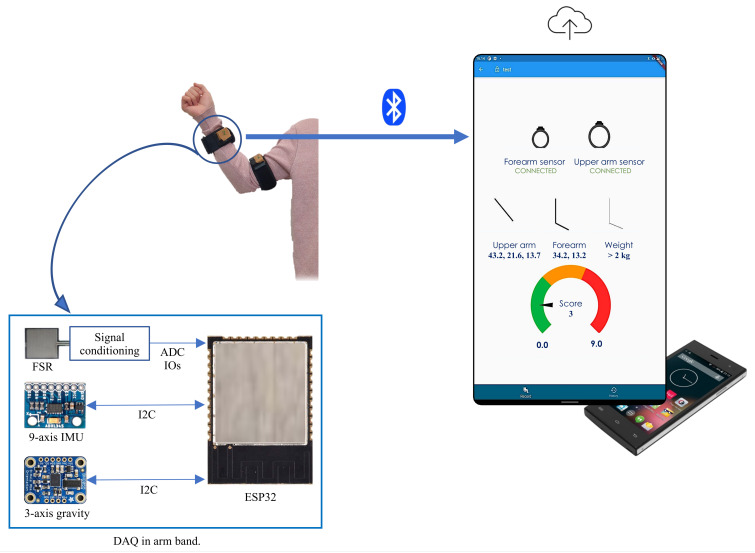
System hardware and software overview.

A portable and easy-to-deploy multi-modal digital upper limb assessment system, DULA, has been developed for automatic musculoskeletal risk assessments as shown in Figure 5. DULA continuously tracks and records upper limbs motion as well as upper arm muscle activity. The system generates single-digit risk assessment scores, ranging from 1 to 9, which indicates the risk of developing MSDs. A lower score means that human postures for carrying out physical tasks are appropriate, implying that no change in the working pattern is required, while a higher score corresponds to higher chances of developing MSDs implying that the working pattern should be immediately changed. The system also stores human motion data and muscle activity data in Google Firebase for visualization and in-depth analysis by medical experts.

### 3.1. Hardware

The hardware of the designed system consists of wireless, battery-powered, multi-modal sensor bands and a mobile phone or tablet. The bands are connected to mobile/tablet applications through Bluetooth. Each sensor band has various sensors, e.g., an IMU consisting of a gravity sensor, gyroscope, linear and angular accelerometers, and eight FSRs. All the sensors provide kinematic information except FSRs, which are used for FMG to determine the weight being lifted by an individual. FSRs are embedded in flexible armbands to have a tight grip over muscles. Data from FSRs are amplified and passed through low pass filters before feeding to analog to onboard digital conversion (ADC) modules, while data from IMU are acquired using the I2C protocol of the ESP32 chip. This chip then sends the data to the mobile phone in real-time through the onboard Bluetooth module.

### 3.2. Software

A mobile application is developed to receive data from sensor bands and to perform the matrix operations for digital upper limb assessment in real-time. On startup, this application guides the user through the procedure of attaching the reference coordinate frames with left and right forearms and upper arms. Once coordinate setup is complete, multi-modal data is acquired, and matrix operations are performed in the background of the mobile application. Risk assessments, limb movements, and muscle usage are graphically shown on the screen. The application has an additional feature of triggering an alarm/notification if an individual is at a high risk of developing MSDs. The application is developed using Flutter and vector_math_64 library is used for matrix operations.

## 4. Results

**Figure 6:**
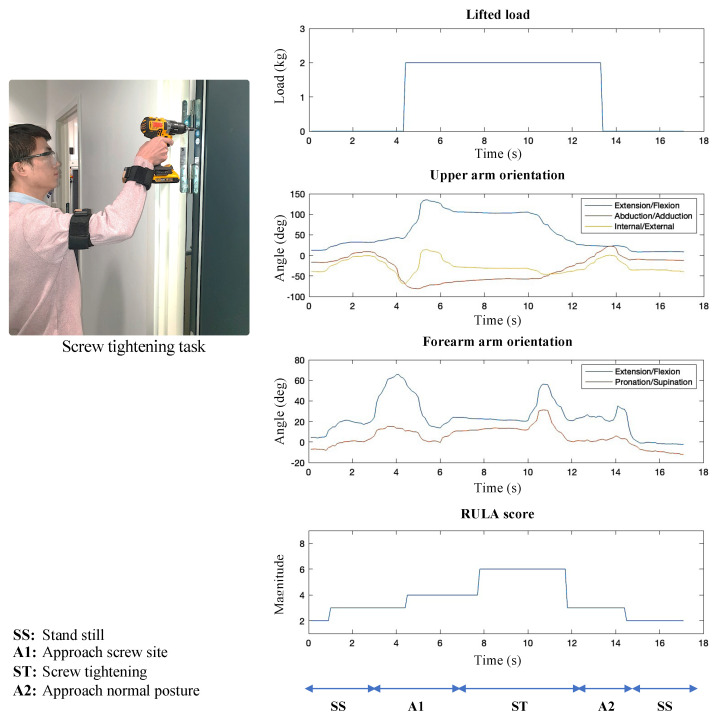
Screw tightening task, corresponding data from the sensors, and RULA score.

With the developed algorithm and system, a DULA score has been generated for the right upper limbs as a test scenario where a person picked an electrical screwdriver from a table and tightened a screw on the wall. Figure 6 shows the body posture for the screw-tightening task and corresponding limb orientations. Angular velocity and gravity data are used only for coordinate frames setup as described in Section 2.2.1 and are not shown here. The orientation of the upper arm and forearm, upper arm abduction, forearm working out to the side of the body or across body mid-line, wrist twist, and carried weight are the factors considered to digitally fill up the RULA score chart. The DULA score is generated in accordance with Figure 1, leading to the generation of the final RULA score according to the method described in Reference [9]. Figure 6 shows that the designed experiment consists of five major movements. The DULA score for each phase shows that there are no ergonomic risks associated with standing still (SS), while approach phases (A1 and A2) show DULA scores of 3 and 4, respectively, indicating that these postures and movements do not carry high risks for developing MSDs.

**Figure 7:**
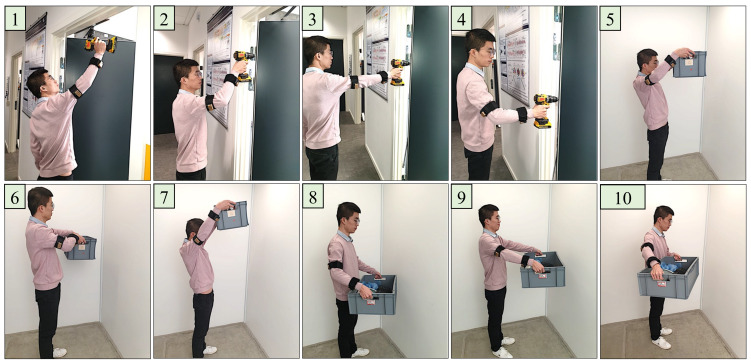
Ten scores for ten different postures and lifting three different weights.

Correlating the DULA score for the screw tightening task with Table 2 indicates that the working routine will soon require changes, and the long time exposure in carrying out this task in the same pattern will certainly lead to developing MSDs. Hence investigations to improve the task procedure or workstation are suggested. To validate the proposed methodology, a web-based ergonomic assessment tool for RULA [33] is used to manually input upper limb postures to generate corresponding RULA scores. Ten different body postures as shown in Figure 7, and three different weights (1.5 kg, 6 kg, and 12 kg) are set for an individual, and RULA scores from the web-based tool and from the DULA system are compared as shown in Table 3. The verification of DULA is ensured by matching the generated scores, as the difference in the RULA scores was 0.3 ± 0.49.

## 5. Discussion

RULA is well developed ergonomic assessment tool, effective for MSD prevention. In this study, a new system, named DULA, is designed for digital rapid upper limb assessment for MSD early prevention and intervention. The system can be used by physical workers and can be easily deployed in any working setup. In addition, the system has the potential to be used with assistive devices, e.g., wearable exoskeletons [30], for their performance assessment, ensuring their effective use. For ergonomic risk assessments in industrial environments, different studies have evaluated ergonomic risks by recording workers’ movements, through questionnaires, or manual analysis by medical experts, which are time-consuming and delicate evaluations. The DULA system effectively addresses these challenges using wearable sensors linked with a mobile application and performing the risk assessment in real-time. However, RULA may not be suitable for assessing ergonomic risks associated with very complex or specialized tasks, which may require a more tailored approach to indicate a correct risk evaluation and would also require more wearable sensor bands. Nevertheless, RULA combined with fuzzy logic has the potential to indicate a better risk level. The same methodology can be further enhanced by tracking and scoring lower limbs, wrists, and necks.

It has to be noted that the risk of developing MSDs is also influenced by various body-related factors, such as age, height, weight, and any comorbidities that the worker may have. RULA provides quick and valuable insights into the potential for injury or discomfort associated with a particular job or task. However, it has limitations in considering the time for performing the task and environmental conditions, e.g., temperature, lighting, noise, etc. Upper arm muscles are responsible for lifting weight. Their role is particularly significant when there is elbow flexion. However, when the elbow is not flexed, then instead of upper arm muscles, weight is carried by bones or by shoulder muscles. In such circumstances, FSRs over bicep muscles are not helpful in identifying the right value of the picked load. Regarding the measurement of muscle activation to determine the lifted load, limitations occur when FSRs become saturated or FSRs experience external force. Consequently, FMG data becomes erroneous. Anti-saturation is required for the post-processing of FMG data. Another limitation is related to an inherent singularity with Euler angles. Euler angles have the intrinsic property that they undergo singularity when the rotation about the middle axis makes the first and third axes parallel to each other.

## 6. Conclusions

The proposed system, DULA, digitally records and automatically analyzes upper limb motions and muscle activities to determine the RULA score for the upper limbs without any intervention from a medical expert. Preliminary results have shown that DULA comprehends RULA. For the ten different postures and three different weights (1.5 kg, 6 kg, and 12 kg), the difference between RULA and DULA is 0.3 ± 0.49.

DULA is a portable system that can be easily deployed in industry and possesses great potential to protect workers from developing temporary or permanent MSDs through early intervention and prevention. In future research, the following issues will be considered to further enhance the methodology: (a) exploring the impact of external forces on FMG, (b) incorporating the analysis of wrist, neck, and lower limbs to enable comprehensive and automated whole-body posture analysis, and (c) integrating task duration into RULA to enhance the accuracy of the assessment. These research directions hold great potential in advancing the field of workplace ergonomics and contributing to the development of effective interventions to prevent musculoskeletal disorders.

## Figures and Tables

**Table 1:** List of related works.

Reference	Assessment Tool	Data Acquisition Method	Wearable	Non-Obstructive	Load Identification
[10,11,12,13,23,24]	RULA/Modified RULA/SI	Optical/Self-report	No	No	No
[14,15,16]	Muscle activity level	EMG/AnyBody software	Yes	Yes	Yes
[20,21,22]	RULA	DHM/VR/CATIA software	Yes	No	No
[17,19]	RULA/REBA/NIOSH	Inertial	Yes	Yes	No
[18]	Artificial Intelligence based	Inertial and optical	Yes	No	No
Our work	RULA	Inertial	Yes	Yes	Yes

**Table 2:** RULA score, action level, and corresponding action.

RULA Score	Action Level	Action
1–2	1	Acceptable working pattern; no changes are required.
3–4	2	Working pattern may be changed; hence, further investigations are suggested.
5–6	3	Working pattern will soon require changes; therefore, further investigations are needed.
>7	4	Working pattern highly risks health and should be immediately changed.

**Table 3:** RULA and DULA scores.

Posture	DULA	RULA	Posture	DULA	RULA
1	6	6	6	6	6
2	5	5	7	7	6
3	5	4	8	5	5
4	4	4	9	6	6
5	6	5	10	7	7

## Data Availability

Not applicable.

## Abbreviations

The following abbreviations are used in this manuscript:

MSD	Musculoskeletal disorders
RULA	Rapid upper limb assessment
DULA	Digital upper limb assessment
IMU	Inertial measurement unit
F or f	Forearm
U or f	Upper arm
